# Non-contact intracellular binding of chloroplasts *in vivo*

**DOI:** 10.1038/srep10925

**Published:** 2015-06-04

**Authors:** Yuchao Li, Hongbao Xin, Xiaoshuai Liu, Baojun Li

**Affiliations:** 1 State Key Laboratory of Optoelectronic Materials and Technologies, School of Physics and Engineering, Sun Yat-Sen University, Guangzhou 510275, China

## Abstract

Non-contact intracellular binding and controllable manipulation of chloroplasts *in vivo* was demonstrated using an optical fiber probe. Launching a 980-nm laser beam into a fiber, which was placed about 3 μm above the surface of a living plant (*Hydrilla verticillata*) leaf, enabled stable binding of different numbers of chloroplasts, as well as their arrangement into one-dimensional chains and two-dimensional arrays inside the leaf without damaging the chloroplasts. Additionally, the formed chloroplast chains were controllably transported inside the living cells. The optical force exerted on the chloroplasts was calculated to explain the experimental results. This method provides a flexible method for studying intracellular organelle interaction with highly organized organelle-organelle contact *in vivo* in a non-contact manner.

Intracellular organelle interactions play an important role in the maintenance of the cellular activity, as well as in cell degradation and death^1^,^2^,^3^. Direct organelle interactions such as close contact between organelles, known as organelle membrane contact sites, can facilitate organelles communications, coordinate cellular functions, and provide a passage for ions (*e.g*. Ca^2+^) and lipids between organelles^4^,^5^,^6^,^7^. Chloroplasts, are typical organelles in plant cells, that are essential to photosynthesis and plant gene engineering, accordingly, all life on earth depends on chloroplasts^8^,^9^. Investigations of direct contact of chloroplasts have the potential to reveal the mechanisms responsible for photosynthesis and chloroplast signaling^10^. However, it is difficult to investigate direct contact of organelles in living cells owing to the dynamic nature of this process as a result of cytoplasmic streaming. This issue can be solved by studying organelles *in vitro*^11^, or using standard chemical fixation and high pressure freeze fixation to create a static environment inside cells^12^. However, *in vitro* investigations may not reflect the biological activities precisely owing to the complexity and variation of the environment *in vivo*^13^. In addition, the fixation techniques will inevitably damage the cells and cannot provide a native state of living cells^14^,^15^. To study the interaction of organelles more accurately, a non-contact method, that can be applied to manipulate and bind organelles *in vivo* without damage, is highly desired. Living cell imaging and fluorescent protein labels provide useful tools for investigating organelle interactions^10^,^16^. However, these methods require complex devices and additional labels.

Optical manipulation, derived from single-beam optical traps known as optical tweezers, may be an appropriate method for non-invasive and non-contact organelle manipulation. Indeed, optical manipulation has been developed as a powerful tool^17^ and successfully applied to the manipulation of biological samples such as cells^18^, viruses^19^, and DNA^20^. Optical manipulation of a single molecule^21^, organelle^22^,^23^, particle^24^,^25^, and red blood cell^13^ *in vivo* or in living cells has also been reported. Additionally, multiple particles have been trapped and bound via redistribution of incident light fields by the presence of particles^26^,^27^,^28^,^29^. Although optical manipulation has been found to be useful for trapping intracellular organelles in plants cell and fungi^23^,^30^,^31^, *in vivo* patterning ordered intracellular organelle arrays have not been achieved to date. Conventional optical tweezers, which are based on optical microscope by focusing free space laser beam with a high numerical aperture objective and a bulky optical system, have become powerful tools for manipulating single and multiple particles. Conventional optical tweezers assisted methods, such as holographic optical tweezers, can also manipulate larger numbers of particles and organellesvia a spatial light modulator to generate multiple beams^17^,^32^. However, optical tweezers have some limitations resulting from their complicated and expensive steering devices (galvo mirrors, acousto-optic deflectors, spatial light modulators) and optical systems (microscope objectives). Fortunately, optical fiber-based optical tweezers have been demonstrated^33^,^34^,^35^ to be suitable for biological manipulation and optical binding^28^,^36^,^37^. When compared with the optical tweezers, optical fiber probes (OFPs) are more miniaturized, handy, and simpler to operate. In this study, we report a non-contact optical method for intracellular binding of chloroplasts and formation of chloroplast chains in a living plant leaf using an OFP.

## Results

**Figure 1:**
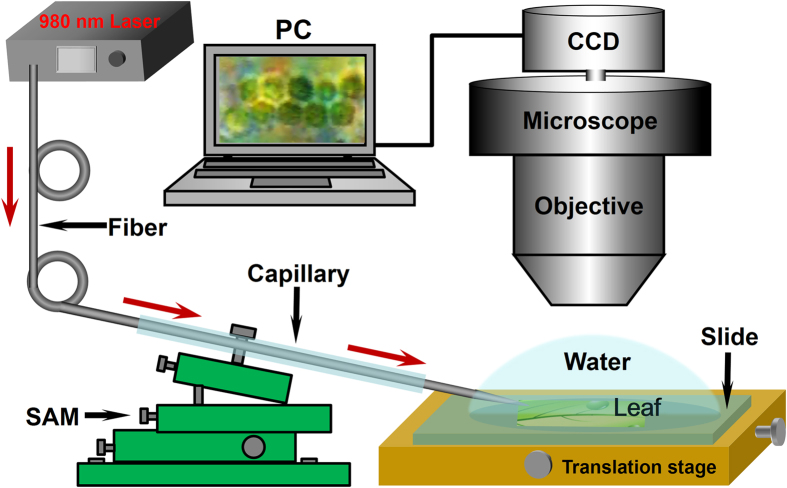
Schematic of experimental setup. An optical fiber probe (OFP) was connected to a 980-nm laser source and the tip was aslant placed above a plant leaf. The red arrows along the fiber indicate the propagation direction of beam. The fiber, was sheathed by a glass capillary and manipulated by a six-axis manipulator. A microscope was used for experimental process observation, while a computer-connected CCD camera was used for image and video capture.

Figure 1 shows the experimental setup. Briefly, a plant leaf was placed flat on a glass slide. To retain its viability, the leaf was immersed in water and the whole plant was wrapped in wet cotton. The tapered end of the fiber was aslant placed above a plant leaf, and manipulated by a six-axis microstage (SAM) with a precision of 50 nm. The distance between the fiber probe and the leaf could be controllably manipulated by the SAM. A 100 × microscope objective (Union, HISOMET II-DH II) with a numerical aperture of 0.73 was used for observation of the experimental process, while a computer-connected CCD camera was used for image and video capture. The total magnification in the field of view of a PC screen is 1000× . The sample was illuminated from the bottom of the sample by a lamp.

**Figure 2:**
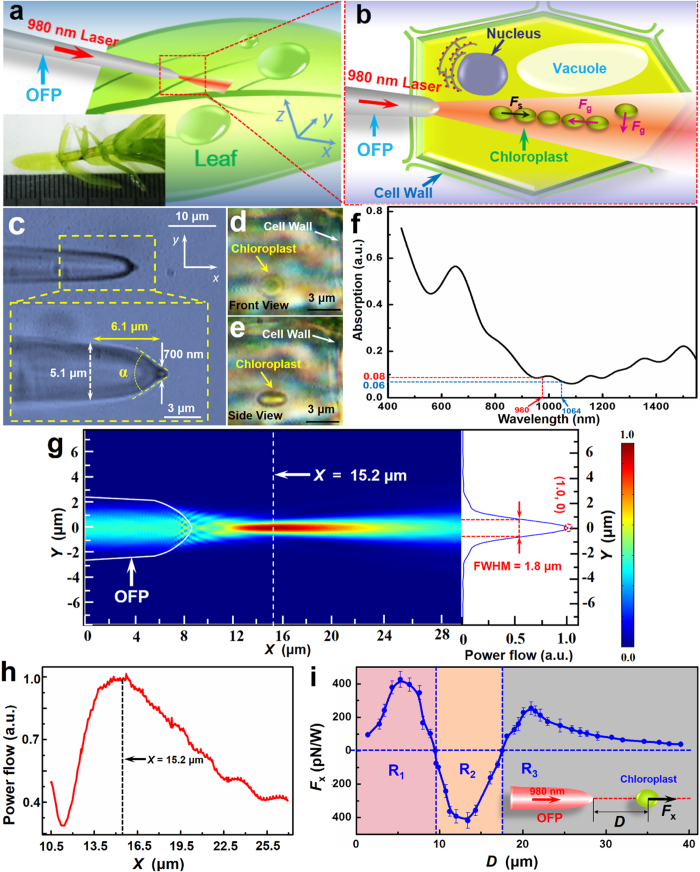
Schematic, optical microscopy image, absorption spectrum, optical power distribution, and calculated optical force. (**a**) Schematic of an OFP is aslant placed above the plant leaf with a laser at 980 nm in the OFP. The inset shows a living plant (*Hydrilla verticillata*) on a glass slide. (**b**) Schematic of optical binding of chloroplasts inside a plant cell shows a row of chloroplasts confined in the optical axis and bound to each other, resulting from the cooperation of *F*_*g*_ and *F*_*s*_. (**c**) Optical microscopy image of the OFP used in the experiment showing an abruptly tapered shape with a diameter that decreased from 5.1 μm to 700 nm within 6.1 μm in the *x* direction. The taper angle α of the OFP was 74°. (**d**) Front view of a chloroplast inside a plant cell. (**e**) Side view image of a chloroplast inside a plant cell. (**f**) Absorption spectrum of plant cells (*Hydrilla verticillata*) in the visible and near infrared wavelengths. (**g**) Simulated optical power flow distribution. The right panel is the normalized power flow distribution at the focal plane (*x *= 15.2 μm) in the *y* direction. (**h**) The normalized power flow distribution along the OFP axis in the *x* direction at *y *= 0 μm. (**i**) Optical force (*F*_*x*_) exerted on a chloroplast in *x* direction as a function of distance *D* to the end of the OFP. The inset is the schematic for calculation of *F*_*x*_.

Figure 2a shows a schematic of an OFP above a plant leaf with an approximately 3-μm gap between the fiber tip and the leaf for optical binding of chloroplasts in a plant cell. The inset of Fig. 2a shows the submersed aquatic angiosperm used in this experiment, *Hydrilla verticillata*, which is commonly employed for observation and study of chloroplasts. As shown in Fig. 2b, with a laser at 980 nm launched into the fiber, the chloroplasts in the mesophyll cells were trapped and bound together, followed by formation of a chain of chloroplasts. It should be noted that a 980-nm laser was used because most living matter exhibits low absorption of this wavelength^38^,^39^.

Figure 2c shows the optical microscopy image of the OFP, which was fabricated by heating and drawing a commercial single-mode optical fiber. The shape of the fiber tip can be modified by controlling the drawing speed. The diameter of the OFP gradually decreased from 8.0 to 5.7 μm within a length of 20.6 μm, then abruptly decreased from 5.1 μm to 700 nm within 6.1 μm. The taper angle of the OFP was *α *= 74°. Figure 2d is the front optical microscopic image view of the chloroplast in the living plant while Fig. 2e is the side view. The chloroplast was ellipsoid with diameters of the major and minor axis are 2.3 ± 0.1 μm and 1.2 ± 0.1 μm, respectively. Figure 2f shows the absorption spectrum of the plant cells. The results in this figure show that the absorption of 980 nm light was 8%, which was slightly higher than that of 1064 nm light (6%). Thus, both of the two wavelengths are suitable for optical tapping in plant cells.

To show the optical binding ability of the OFP, normalized optical power flow distribution output from the OFP was simulated by a finite-element method using Comsol Multiphysics 4.4 and is shown in Fig. 2g. In the simulation, the power of the 980-nm laser was set to 35 mW and the refractive indices of the OFP and water were set to 1.44 and 1.33, respectively. Benefiting from the abruptly tapered shape, the laser beam is highly focused near the end of the fiber tip. Figure 2h shows the power flow distribution along the OFP axis in the *x* direction (*y* = 0).The focal plane with the strongest light intensity was at *x* = 15.2 μm (*i*.*e*. 6.8 μm away from the fiber tip). The right panel of Fig. 2h shows the optical power flow at the focal plane (*x* = 15.2 μm) in the *y* direction. The full width at half maximum (FWHM) was 1.8 μm, which was comparable to the size of chloroplasts so that they could be confined in the optical axis of the OFP. Figure 2i shows the calculated optical forces (*F*_*x*_) exerted on the chloroplast along the OFP axis as a function of axial distance *D* to the end of the OFP. The *F*_*x*_ consists of gradient force *F*_*g*_, which traps chloroplasts towards the high-intensity region and scattering force *F*_s_, which drives chloroplasts along the direction of light propagation. The moving velocity (*V*) of the chloroplasts is determined by the equilibrium of the optical force (*F*_*x*_) and the Stokesian drag force (*F*_drag_), therefore, the optical force *F*_*x*_ can be calculated according to Stokes’ law^40^:


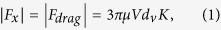


where μ is the cytoplasmic viscosity of the plant cell which is estimated to be 3.0× 10^−2^ Pa·s (see details in Supplementary Information) at room temperature^41^,^42^, and *d*_v_ is defined as the diameter of a sphere with the same volume as the ellipsoidal chloroplast. The measured value of *d*_v_ = 1.8 ± 0.1 μm. And *K* is the shape factor^43^:


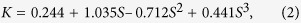


where *S* is the surface area of the chloroplast. For the chloroplasts used in this experiment, the estimated *K* is 1.15 ± 0.09.

The streaming force, generated by cytoplasmic streaming, will influence the results of the calculated optical force. In this study, two methods were applied to minimize the influence of streaming force. (1) Because the cytoplasmic streaming was mainly in the *y* direction in the central section of the cell, this section was selected to measure the optical force in the *x* direction. (2) The experiment was performed in a cell with a relatively slow cytoplasmic streaming velocity (average velocity below 0.4 ± 0.1 μm/s). Based on these two methods, the streaming force was quite small in the *x* direction relative to the optical force and had no obvious influence on the results.

As shown in Fig. 2i, for a region of *D* < 9.5 μm (R_1_), *F*_*x*_ > 0, indicating that *F*_s_ is larger than *F*_*g*_. Thus, the chloroplasts in this region will be propelled away from the OFP. For the chloroplast in 9.5 μm < *D* < 17.8 μm (R_2_), *F*_*x*_ < 0, indicating that the dominant force in this region is *F*_*g*_ and the chloroplasts will be trapped. For *D* > 17.8 μm (R_3_), *F*_*x*_ > 0, indicating that the chloroplasts in this region will be propelled away. When *D* > 20.9 μm, *F*_*x*_ is gradually decreased with increasing *D* because of the decrease in light intensity.

**Figure 3:**
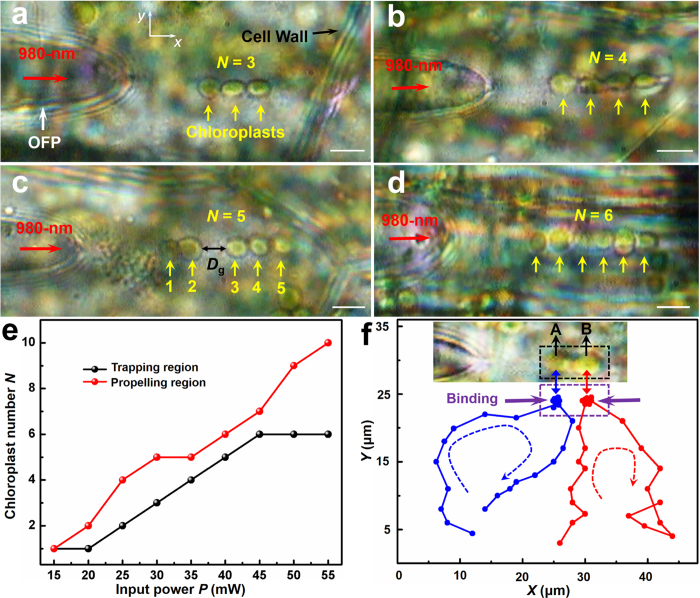
Optical binding of chloroplasts. (**a-d**) Optical microscopy images of chains of three, four, five, and six chloroplasts formed at input optical powers of *P* = 30, 35, 42, and 50 mW, respectively. The scale bar is 5 μm. (**e**) Chloroplast numbers (*N*) trapped in chains as a function of input optical power. (**f**) Trajectory of two chloroplasts inside a plant cell. The inset shows the binding of two chloroplasts. Position data were obtained every 2 s.

With a laser beam of 980 nm launched into the OFP, the chloroplasts beside the optical axis of the OFP were trapped and confined to the axis by the transverse *F*_*g*_ (in the *y* direction). The chloroplasts were then bound tightly and formed a chloroplasts chain by the longitudinal *F*_*g*_ (in the *x* direction). Figures 3a–d show chains of three, four, five, and six chloroplasts formed in the plant cells at input optical powers *P* = 30, 35, 42, and 50 mW, respectively. As shown in Fig. 3c, there was a gap with distance *D*_g_ = 4 μm between the second and the third chloroplasts, because the first two chloroplasts were trapped by *F*_*g*_ and the latter three were propelled away by *F*_*s*_. Figure 3e shows the relationship between the largest number of chloroplasts trapped in a chloroplasts chain and input optical power. The results clear show that the number of chloroplasts in the chain in the propelling region (R_3_) increased with increasing input optical power, because of the increasing optical force. However, in the trapping region (R_2_), the largest chloroplast numbers increasing with the increasing optical power was observed when *P* < 45 mW. When *P* > 45 mW, the largest chloroplast numbers no longer changed with increasing optical power, and the maximuml number of chloroplasts was six. The size of the trapping region had a maximum value that was unchanged with increasing optical power. It should be noted that, the trapping region could be expanded by increasing the taper angle α of the OFP. Figure 3f shows a trajectory of two chloroplasts (marked A and B) inside a plant cell. Before binding, chloroplasts A and B moved with average velocities of 1.3 ± 0.2 and 1.0 ± 0.1 μm/s, respectively, because of cytoplasmic streaming and Brownian motion. When moving to the trapping region of the OFP, the two chloroplasts were trapped and bound by the 980 nm laser with a power of 25 mW for about 20 s. The binding positions fluctuated in response to the environmental fluctuations and Brownian motion. Further experiments demonstrated that, chloroplasts A and B moved away from the binding position after the laser was turned off with average velocities of 1.3 ± 0.1 and 1.0 ± 0.1 μm/s, respectively.

**Figure 4:**
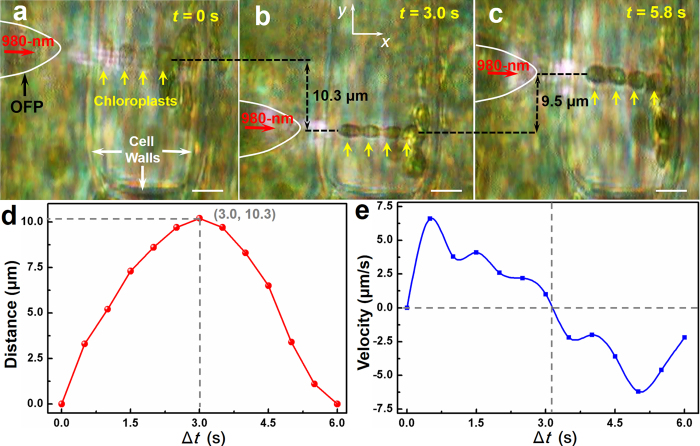
Optical transport of chloroplasts chain in the plant cell. (**a-c**) Optical microscopy images of the chain during transportation in the y direction. The scale bar is 5 μm. (**a**) *t* = 0, a chain of four chloroplasts was formed in the center of the cell at an optical power of 35 mW. (**b**) *t* = 3.0 s, the chain was transported 10.3 μm in the –*y* direction. (**c**) *t* = 5.8 s, the chain was transported 9.5 μm in the + *y* direction. (**d**) The distance the chain moved in the *y* direction as a function of time Δ*t* at an optical power of 35 mW. The peak movement of 10.3 μm occurred at Δ*t* = 3.0. (**e**) The velocity of the chain in the *y* direction as a function of Δ*t.*

The formed chloroplasts chain could be flexibly transported inside cells by moving the OFP. Figure 4 shows the transport of a chloroplasts chain in the *y* direction. At *t* = 0, a chain with four chloroplasts was formed in the center of the cell with an optical power of 35 mW and stably confined in the optical axis of the OFP by the transverse *F*_*g*_ (Fig. 4a). By moving the OFP in the –*y* direction with an average velocity of 3.4 ± 0.2 μm/s, the chain was transported 10.3 μm in the –*y* direction within 3.0 s (Fig. 4b). The chain was then transported in the + *y* direction by moving the OFP. In addition, the chain was transported 9.5 μm in the + *y* direction within 2.8 s at an average velocity of 3.4 ± 0.1 μm/s (Fig. 4c). The experiment also showed that, no chloroplasts escaped from the chain during transportation. To maintain a stable chloroplasts chain during the transporting process, at an optical power of 35 mW, the maximum moving velocity (*V*_m_) of the OFP is 7.5 ± 0.2 μm/s. Since the optical force increases with increasing optical power, a higher *V*_m_ can be achieved by increasing the optical power. For example, at an optical power of 60 mW, the maximum moving velocity *V*_m_ reached 13.1 ± 0.2 μm/s. Figure 4d shows the transported distance while Fig. 4e shows the moving velocity of the chain as a function of moving time Δ*t* at an optical power of 35 mW. It is important to note that, transverse (in the *y* direction) cytoplasmic streaming caused the chain to rotate around the optical axis during transport in the cell.

**Figure 5:**
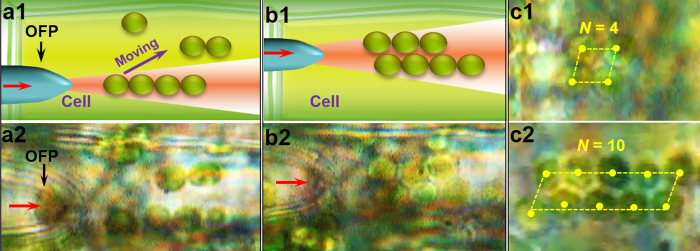
2D organelle-organelle contact. (**a,b**) Schematics and microscopic images of the 2D organelle-organelle contact process. (**c**) Two rows of chloroplasts with different numbers of chloroplasts.

Because the developed method was shown to be capable of inducing chloroplasts transport, one-dimensional organelle-organelle contact was extended to two-dimensional (2D) organelle-organelle contact by binding two rows of chloroplasts. Figures 5a1 and 5b1 show the 2D organelle-organelle contact process, while Figures 5a2 and 5b2 show the experimental microscopy images. By launching a laser beam of 980 nm with an optical power of 35 mW, a four-chloroplast chain was firstly formed near the tip of the OFP (Fig. 5a) and then the chain was moved to approach other chloroplasts by moving the OFP with an average velocity of 3 ± 0.2 μm/s. The chloroplasts beside the first chain were trapped by transverse *F*_*g*_ (in the *y* direction) and formed another chain close to the first chain. As a result, the two chains were bound to each other and a 2D chloroplasts array was formed (Fig. 5b). Figures 5c1 and 5c2 show that, two rows of chloroplast with chloroplasts numbers *N* = 4 and 10 were formed at optical powers of 30 and 45 mW, respectively. To determine the stability of the 2D organelle-organelle contact during cytoplasmic streaming, an additional experiment was conducted to maintain the 2D chloroplasts array with chloroplast numbers *N* = 6. The results indicated that, the 2D chloroplasts array was stably maintained for over 10 min until the laser was turned off at a cytoplasmic streaming velocity of 0.9 ± 0.1 μm/s. The yellow dots in Fig. 5c indicate the centers of the chloroplasts that formed a parallelogram.

## Discussion

Chloroplast transport is known to be a natural phenomenon in living plant cells that enables chloroplasts redistribution, which can improve photosynthesis efficiency and avoid damage caused by strong light. However, this process normally takes over an hour in living cells^44^. Rapid and effective redistribution of chloroplasts can be achieved using the developed optical method to controllably transport chloroplasts inside living cells. Furthermore, the method described herein can be used to transport chloroplasts for contact with other organelles such as the mitochondria and endoplasmic reticulum, to enable investigation of cross-talk between chloroplasts and other organelles.

The well-regulated arrays of chloroplasts could be formed by the developed method. Interestingly, such well-regulated bio-material array structures exist in nature^45^. Artificial well-regulated arrays of cells on micro-patterned material surfaces have been used to study cell-cell contact^46^. Similarly, organelle-organelle contact, especially exchange of information and material through organelle membrane contact sites, could be investigated by patterning well-regulated 2D chloroplasts arrays in living cells using this optical method.

For application of this method *in vivo*, it is essential that the cell be viable during investigation of intracellular organelle contact. To test the plant cell viability, a further experiment was carried out *in vivo*. Cytoplasmic streaming indicates a healthy living cell, and chloroplasts movement that resulted from cytoplasmic streaming, can be used to verify the cell viability^47^. Repeated experimental observations indicated that the viability of plant cells was not influenced, by radiation for 20 min with an optical power of 65 mW. Although chloroplasts were used as objects for optical binding and organelle-organelle contact, the non-contact method would also be effective for other intracellular particles with a larger refractive index than that of the surrounding medium, such as mitochondria and chromosomes.

**Figure 6:**
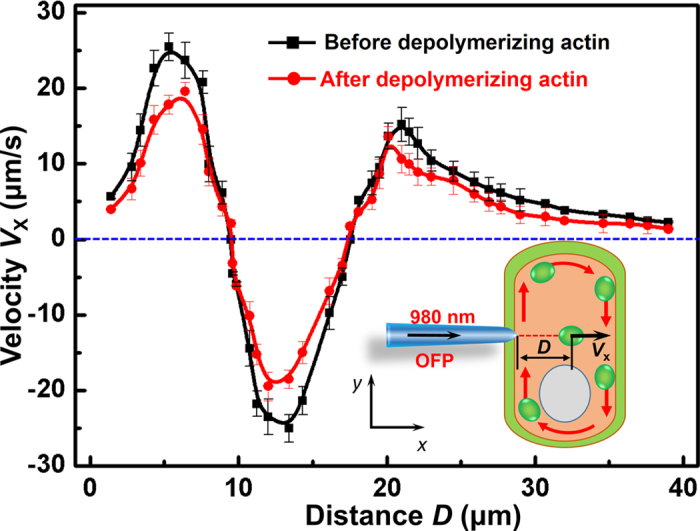
Velocity of chloroplasts before and after depolymerizing actin. Velocity of chloroplast movement in the *x* direction as a function of distance between the chloroplasts and the optical fiber probe before and after depolymerizing actin. The red arrows along the cell wall in the inset indicate the direction of cytoplasmic streaming.

It should be noted that the optical force was roughly approximated in the experiments. Measuring optical force accurately *in vivo* is challenging because of the complex environment inside living cells. These challenges include difficulties in estimating the cytoplasmic viscosity and influences of the elastic component in cytoplasm. Specifically, the complex spatial distribution of components inside living cells, makes it much more difficult to measure cytoplasmic viscosity accurately *in vivo*. Therefore, several parameters (*e.g.* the correlation length *ξ*) of the particular cells in our experiments were estimated based on a previously described model^42^. Additionally, the cytoplasm is known to be viscoelastic because of the presence of cytoskeletal filaments (actin in this case) that hinder organelle movement. To simplify the issue, the cytoplasm was assumed to be a Newtonian fluid in the present study. The more accurate measurement of optical force should consider the influence of the elastic component of the cytoplasm. To exclude the influence of actin, an additional experiment was conducted in which actin was depolymerized and cytoplasmic streaming was stopped to measure the velocity and optical force of the chloroplast. Specifically, actin was depolymerized by immersing leaves in 5 μM latrunculin B solution for 9 min at 25 °C. Figure 6 shows the velocity of chloroplasts in the *x* direction as a function of the distance between the chloroplasts and the optical fiber probe before and after depolymerizing actin. It can be seen that the results of velocity before depolymerizing actin and after depolymerizing actin are slightly different. Since optical force is proportional to velocity, there was no obvious influence on optical force. Accordingly, it can be inferred that the elastic component has no obvious influence on the results and the optical force was dominant in the experiments. It should be noted that, the slight increase in velocity after depolymerization might have been caused by decreased cytoplasmic viscosity^48^.

## Conclusion

In summary, we demonstrated an optical method to induce non-contact intercellular binding of chloroplasts *in vivo* using an optical fiber probe. With launched optical powers varying from 30 to 50 mW at 980 nm, stable chloroplast chains with the chloroplast numbers from 2 to 6 were formed in living plant cells. Moreover, the chloroplast chains could be controllably transported inside the cells, and formed into 2D chloroplast arrays with different chloroplast numbers. The viability of the plant cells was also evaluated after increasing the optical power to 65 mW and subjecting the cells to 20 min of radiation. The experiments indicated that, the method had no effect on cell viability. This non-invasive and non-contact method of organelle binding and manipulation will be useful for biological and biochemical research *in vivo*, especially for investigation of signal transduction and communication between intracellular organelles via organized organelle-organelle contact.

## Additional Information

**How to cite this article**: Li, Y. *et al.* Non-contact intracellular binding of chloroplasts *in vivo*. *Sci. Rep.* **5**, 10925; doi: 10.1038/srep10925 (2015).

## Supplementary Material

Supplementary Information
