# Supplementary material for: Non-contact intracellular binding of chloroplasts in vivo
Source: Sci Rep. 2015 Jun 4;5:10925. doi: 10.1038/srep10925 (PMC4455249; doi:10.1038/srep10925)
Supplement: Supplementary Information [file srep10925-s1.pdf]

# Supplementary Information

## Non-contact intracellular binding of chloroplasts *in vivo*

Yuchao Li, Hongbao Xin, Xiaoshuai Liu, and Baojun Li*
*State Key Laboratory of Optoelectronic Materials and Technologies, School of Physics and Engineering, Sun Yat-Sen University, Guangzhou 510275, China*
*Email: stslbj@outlook.com

### 1. Cytoplasmic viscosity of plant cell

The cytoplasmic viscosity of plant cell measured by fluorophores (kiton red, see details in Ref. [1]) as probe particles, which is defined as nanoscopic viscosity, is $\eta_n = 1.3 \times 10^{-3}$ Pa·s [1]. For microparticles, the viscosity can be corrected to macroscopic viscosity $\eta_m$ [2]. The relation between the macroscopic viscosity $\eta_m$ and the nanoscopic viscosity $\eta_n$ is

$$\eta_m = \eta_n \exp\left[(R_{eff}/\xi)^a\right], \quad (1)$$

where $R_{eff}$ is the effective hydrodynamic radius, $\xi$ is the correlation length, and $a$ is a constant of order of 1. $\xi$ and $a$ are estimated to be $\xi = 5 \pm 4$ nm and $a = 0.5 \pm 0.1$. The effective hydrodynamic radius $R_{eff}$ is defined as

$$R_{eff}^{-2} = R_h^{-2} + r_p^{-2}, \quad (2)$$

where $R_h$ is the hydrodynamic radius and $r_p$ is the radius of the probe particle. $R_h$ can be expressed as

$$R_h = L/(2s - 0.19 - 8.24/s + 12/s^2), \quad (3)$$

where $s = \text{In}\,(L/r)$, $L$ and $r$ are the length and the radius of the actin filament, respectively. $L$ is estimated to be 300 nm [3], while $r$ is estimated to be 7 nm [2]. The viscosity is calculated as $\eta_m = 3.0 \times 10^{-2}$ Pa·s, which is reasonable compared to the reported results of $4.4 \times 10^{-2}$, $2.4 \times 10^{-2}$, and $5 \times 10^{-2}$ Pa·s for HeLa, Swiss 3T3, and an amoeba *Dictyostelium discoideum* cells, respectively [2,4].

### References

1. A. Srivastava & G. Krishnamoorthy, "Cell type and spatial location dependence of cytoplasmic viscosity measured by time-resolved fluorescence microscopy". *Arch. Biochem. Biophys.*, **340**, 159–167 (1997).
2. T. Kalwarczyk *et al.* "Comparative analysis of viscosity of complex Liquids and cytoplasm of mammalian cells at the nanoscale". *Nano Lett.*, **11**, 2157–2163 (2011).
3. E. Dauty & A. S. Verkman, "Actin cytoskeleton as the principal determinant of size-dependent DNA mobility in cytoplasm: a new barrage for non-viral gene delivery". *J. Biol. Chem.*, **280**, 7823–7828 (2005).
4. D. Arcizet, B. Meier, E. Sackmann, J. O. Rädler, & D. Heinrich, "Temporal analysis of active and passive transport in living cells". *Phys. Rev. Lett.*, **101**, 248103 (2008).
